# Molecular genetics in adult-onset Still’s disease: next-generation sequencing in 24 patients and literature review

**DOI:** 10.3389/fimmu.2024.1474271

**Published:** 2024-11-14

**Authors:** Diana Prieto-Peña, Eztizen Labrador-Sánchez, Rafael B. Melero-González, Fred Antón-Pagés, Natalia Palmou-Fontana, Carmen Alvarez-Reguera, Nerea Paz-Gandiaga, Ricardo Blanco

**Affiliations:** ^1^ Rheumatology, Hospital Universitario Marqués de Valdecilla, Santander, Spain; ^2^ Immunopathology Research Group, Instituto de Investigación Marqués de Vadelcilla (IDIVAL), Santander, Spain; ^3^ Rheumatology, Hospital Unversitario San Pedro, Logroño, Spain; ^4^ Rheumatology, Complejo Hospitalario Universitario de Vigo, Vigo, Spain; ^5^ Rheumatology, Complejo Asistencial de Segovia, Segovia, Spain; ^6^ Department of Genetics, Hospital Universitario Marqués de Valdecilla, Instituto de Investigación Marqués de Vadelcilla (IDIVAL), Santander, Spain

**Keywords:** genetics, adult-onset Still’s disease, NGS, molecular genetic techniques, autoinflammatory diseases

## Abstract

**Objective:**

Next-generation sequencing (NGS) panels are increasingly used for the diagnosis of monogenic systemic autoinflammatory diseases (SAIDs). However, their role in patients with adult-onset Still’s disease (AOSD) remains unknown. This study aims to assess the usefulness of NGS panels in AOSD patients to improve diagnosis and management of the disease.

**Methods:**

This observational, multicenter study included all patients with AOSD diagnosis who underwent NGS panel testing in northern Spain. Clinical manifestations, laboratory parameters, complications, and therapeutic responses were recorded.

**Results:**

A total of 24 patients (16 men, 8 women) with an average age of 42.2 ± 17.9 (mean ± SD) years, in whom NGS was performed, fulfilled the Yamaguchi and/or Fautrel criteria for AOSD. The most common symptoms, apart from fever, were skin rash (75%), asthenia (91.7%), and articular manifestations (91.7%). All patients had elevated acute-phase reactant levels and hyperferritinemia. Almost all patients received oral glucocorticoids as initial therapy. Conventional disease-modifying antirheumatic drugs (cDMARDs) were used in 17 (70.8%) patients and biologic therapy in 13 (54.1%) patients. Genetic variants were observed in 5 (20.8%) patients. None of them were classified as pathogenic. Variants of uncertain significance (VUS) were identified in *NOD2* (c.2104C>T and c.2251G>A), *TNFRSF1A* (c.224C>T), *TNFAIP3* (c.1939A>C), and *SCN9A* (c.2617G>A). Atypical manifestations and/or therapeutic refractoriness were observed in patients carrying genetic variants, except for one patient with the *TNFAIP3* VUS. Four out of five patients with VUS had a severe and refractory course of the disease and required biologic therapy.

**Conclusion:**

NGS was useful to rule out the presence of pathogenic genetic variants related to other SAIDs and to detect VUS that may help identify patients at risk for atypical and severe manifestations and poor response to conventional therapy.

## 1 Introduction

Adult-onset Still’s disease (AOSD) is a rare systemic inflammatory disease characterized by intermittent fever, arthritis, and evanescent skin rash in the absence of infection, malignancy, or rheumatological disease (1–4). It usually occurs in young adults with a bimodal age distribution, the first peak between 15 and 25 years and the second between 36 and 46 years. The clinical course varies from one individual to another, ranging from benign and self-limited forms to severe complications such as macrophage activation syndrome (1–4).

Although the etiopathology of AOSD remains unknown, growing evidence supports that AOSD is an autoinflammatory disorder where the innate immune system plays a pivotal role (1). The main hypothesis states that, in patients with some genetic predisposition, several external factors, such as infectious agents and other environmental factors, activate innate immune cells through Toll-like receptors (TLRs), leading to an abnormal response in both innate and adaptive immunity with an overproduction of cytokines, mainly IL-1b, IL-18, and IL-6 (1–4).

AOSD is considered a polygenic disease in contrast to other systemic autoinflammatory diseases (SAIDs) that are related to specific genetic mutations, such as the familial Mediterranean fever syndrome (FMF), TNF receptor-associated periodic fever syndrome (TRAPS), cryopyrin-associated periodic syndrome (CAPS), hyper IgD syndrome (HIDS), or Yao syndrome. Although AOSD shares common clinical and pathogenic features with these SAIDs, its genetic profile seems to be different. The presence of polymorphisms in genes encoding innate immunity-associated factors has been reported in AOSD patients (5–9). However, a unique association with HLA genes (10–13), not observed in other SAIDs, suggests the involvement of the adaptative immune system.

Molecular genetic analysis has become an excellent diagnostic tool for most SAIDs (14–19). However, it is not routinely used in patients with AOSD suspicion despite the fact that it could be very useful in differentiating it from other SAIDs (20, 21). In this regard, experts in the field call for a revision of the current classification criteria for AOSD that include the exclusion of other periodic fever syndromes with the aid of molecular genetics testing (22). Given that AOSD is considered a polygenic disease, the use of molecular genetic techniques targeting specific genes, such as Sanger sequencing, has been more restricted compared to its application in monogenic SAIDs. In this regard, next-generation sequencing (NGS) represents a particularly valuable tool for AOSD. NGS markedly outperforms Sanger sequencing in terms of scalability, speed, cost efficiency, sensitivity, versatility, and clinical applicability. Furthermore, NGS facilitates the development of disease-specific gene panels. Its high sensitivity enables the detection of low-frequency variants, including somatic mutations and mosaicisms, which are often challenging to identify with Sanger sequencing. Additionally, the capability for deep coverage with NGS enhances the precision of variant detection, which is crucial for investigating complex diseases and identifying rare or novel sequence variants.

Scarce studies have focused on the role of molecular genetics analysis in AOSD (22). Our study aimed to analyze the results of NGS in our cohort of patients with AOSD and to conduct a literature review on the potential value of molecular genetic techniques to improve diagnosis and differentiate AOSD from other SAIDs.

## 2 Patients and methods

### 2.1 Study design

We conducted an observational, multicenter study in all patients with AOSD diagnosis in whom NGS analysis was performed.

Patients were diagnosed with AOSD at the rheumatology or autoimmune units of four national referral centers. AOSD diagnosis was based on the Yamaguchi (23) and/or Fautrel (24) criteria. Data were gathered and analyzed according to an agreed protocol. All data were stored in a computerized database. All the procedures were carried out according to the ethical standards of the approved guidelines and regulations, following the Declaration of Helsinki and Good Clinical Practice standards. This study was approved by the Institutional Review Board of Cantabria (IRB N 2022.240). Retrospective data and blood samples have been obtained during routine clinical practice with the written informed consent of the patients.

### 2.2 Clinical and laboratory features

Clinical information and laboratory parameters for AOSD diagnosis and follow-up were obtained from medical records. The following clinical variables were collected: age, sex, ethnicity, duration of symptoms before diagnosis, affected relatives, presence of fever, skin manifestations, mucocutaneous ulcers, arthralgias and/or arthritis, ocular symptoms, cardiopulmonary manifestations, neurologic symptoms, gastrointestinal symptoms, asthenia, constitutional symptoms, lymphadenopathies and/or splenomegaly, and macrophage activation syndrome.

Laboratory parameters included C-reactive protein (CRP) and erythrocyte sedimentation rate (ESR) levels at diagnosis, neutrophilic leukocytosis, anemia, hyperferritinemia, and increased liver transaminase levels. We also reviewed if patients were positive for rheumatoid factor (RF), antinuclear antibodies (ANA), antineutrophil cytoplasmic antibodies (ANCA), and anti-citrullinated protein antibodies (ACPA).

In addition, treatment strategies and responses were also recorded.

### 2.3 NGS high-throughput genotyping

DNA was isolated from peripheral blood samples for the assessment of both quantity and quality and stored at −20°C until analysis. Library preparation for sequencing coding regions of the genes was carried out using Constitutional Panel 17 Mb (CCP17) with Agilent SureSelect Technology™ for Illumina according to the manufacturer’s instructions (NextSeq™). A custom bioinformatics pipeline was developed for the analysis of variants in exonic and +/−10 nucleotide intronic flanking regions with a minor allele frequency (MAF) of less than 1% with respect to genomic reference alignment (hg19). A SAID *in-silico* gene panel was used that included the following: ACP5, ADA2, ADAM17, ADAR, ARPC1B, C17orf62, CARD14, CIB1, COPA, DNASE2, IKBKG, IL-10, IL-10RA, IL-10RB, IL-1RN, IL-36RN, JAK1, JAK3, LACC1, LPIN2, LRP5, MEFV, MVK, NLRC4, NLRP1, NLRP12, NLRP3, NOD2, OTULIN, PLCG2, POLA1, POMP, PROC, PSMA3, PSMB10, PSMB3, PSMB4, PSMB8, PSMB9, PSMG2, PSTPIP1, RBCK1, RC3H1, RIPK1, SLC29A3, TMEM173, TNFAIP3, TNFRSF1A, and WDR1. In addition, the analysis comprised relevant variants in the genes obtained from the following human phenotype ontology (HPO) terms: HP:0001369 Arthritis, HP:0001945 Fever, HP:0000988 Skin rash, and HP:0002829 Arthralgia (https://hpo.jax.org/app/). Complementary explorations for copy-number variants were carried out but did not include complex DNA rearrangements (inversions or translocations), variants within pseudogene regions, or low-level mosaicisms. Classification and annotation of clinically relevant variants were performed based on the recommendations of the American College of Medical Genetics and Genomics (ACMG) (25). The nomenclature used to define the variants follows the criteria of the Human Genome Variation Society (HGVS) (http://www.HGVS.org/varnomen). The selection of the gene panel is based on expert opinion in genetics following a review of scientific literature and specialized databases to identify genes relevant to the association with SAIDs.

### 2.4 Statistical analysis

Statistical analysis was performed using SPSS Statistics for Windows, version 18.0 (SPSS Inc, Chicago, IL, USA). All continuous variables were tested for normality, and results were expressed as mean ± SD or as median and interquartile range (IQR) as appropriate.

## 3 Results

**Figure 1:**
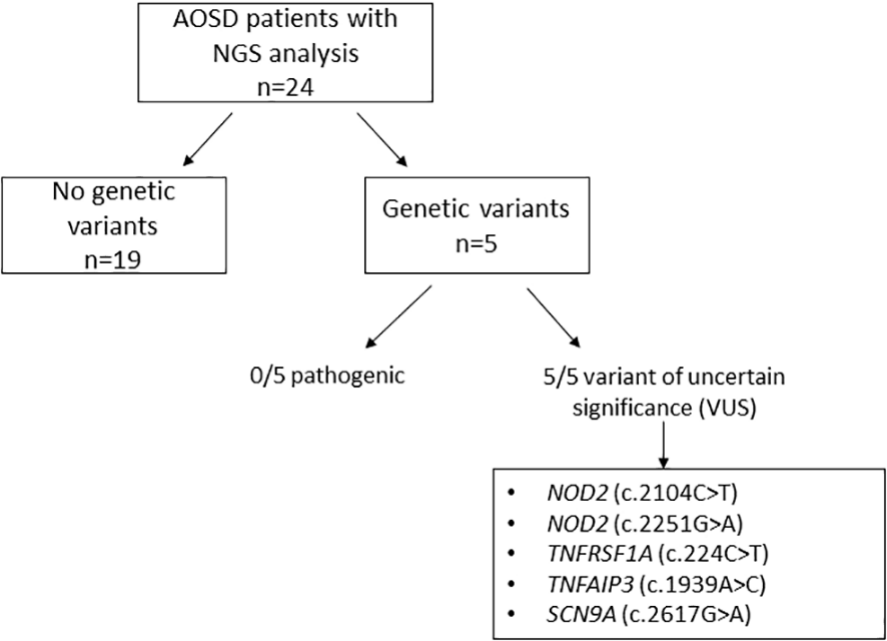
Genetic variants detected by next-generation sequencing in our cohort of 24 AOSD patients.

We identified 24 patients with AOSD diagnosis in whom NGS analysis was performed (**Figure 1**). The main demographic, clinical, and laboratory features are summarized in **Table 1**.

**Table 1:** Main demographic and clinical features of our cohort of AOSD patients.

	AOSD patients (*n* = 24)
Demographic features	
Age at diagnosis (years), mean ± SD	42.2 *±* 17.9
Sex (women), *n* (%)	8 (33.3)
Affected relatives, *n* (%)	1 (4.2)
Ethnicity (Caucasian/Hispanic/African)	22/1/1
Clinical manifestations	
Duration of symptoms after diagnosis (months), median [IQR]	1 (1–10)
Fever, *n* (%)	24 (100)
Skin rash, *n* (%)	18 (75)
Mucocutaneous ulcers, *n* (%)	6 (25)
Odynophagia, *n* (%)	6 (25)
Arthralgia/arthritis, *n* (%)	22 (91.7)
Ocular, *n* (%)	3 (12.5)
Pleuropericarditis, *n* (%)	8 (33.3)
Neurologic, *n* (%)	3 (12.5)
Gastrointestinal, *n* (%)	7 (29.2)
Asthenia, *n* (%)	22 (91.7)
Constitutional syndrome, *n* (%)	13 (54.2)
Lymphadenopathies, *n* (%)	14 (58.3)
Splenomegaly, *n* (%)	6 (25)
Macrophage activation syndrome, *n* (%)	2 (8.3)
Laboratory parameters	
CRP (mg/dL), median [IQR]	10.2 [3.8–24.6]
ESR (mm/1st hour), median [IQR]	70 [45.5–100]
Anemia, *n* (%)	12 (50)
Neutrophilic leukocytosis, *n* (%)	22 (91.6)
Increased liver transaminase levels, *n* (%)	13 (54.2)
Hyperferritinemia, *n* (%)	24 (100)
Immunology tests	
Positive ANA, *n* (%)	2 (8.3)
Positive ANCA, *n* (%)	0 (0)
Positive RF, *n* (%)	2 (8.3)
Positive ACPA, *n* (%)	1 (4.2)
Treatment	
Glucocorticoids, *n* (%)	23 (95.8)
NSAIDs, *n* (%)	14 (58.3)
Colchicine, *n* (%)	7 (29.2)
Conventional DMARDs, *n* (%)	17 (70.8)
- Methotrexate, *n* (%)	17 (70.8)
- Azathioprine, *n* (%)	2 (8.3)
- Leflunomide, *n* (%)	1 (4.2)
Biologic DMARDs, *n* (%)	13 (54.1)
- Anakinra, *n* (%)	8 (33.3)
- Canakinumab, *n* (%)	3 (12.5)
- Anti-TNF, *n* (%)	5 (20.8)
- Anti-IL-6, *n* (%)	6 (25)

### 3.1 Demographic, clinical, and laboratory features

Sixteen men and eight women fulfilled the Yamaguchi criteria for AOSD diagnosis. The mean age at diagnosis was 42.2 ± 17.9 (range 18–70). Most of the patients were Caucasians (91.6%), one patient was African, and another one was Hispanic. Only one patient had affected relatives.

The average time from the onset of symptoms to the disease diagnosis was 1 (1–10) months. All patients had fever and most of them had skin rash (75%), articular manifestations (91.7%), and asthenia (91.7%). Pleuro-pericarditis was reported in eight (33.3%) patients. Lymphadenopathy (58.2%) was more commonly found than splenomegaly (25%). The less common manifestations included ocular symptoms [anterior uveitis (*n* = 2), severe sicca syndrome (*n* = 1)], neurologic manifestations [headache (*n* = 2), posterior leukoencephalopathy (*n* = 1)], gastrointestinal symptoms [abdominal pain (*n* = 6), diarrhea (*n* = 1)], and testicular pain (*n* = 1). Remarkably, macrophage activation syndrome was reported in two patients.

Regarding laboratory parameters at disease diagnosis, all patients had elevated acute-phase reactant levels and hyperferritinemia. The median [IQR] level of CRP was 10.2 [3.8–24.6] mg/dL and ESR was 70 [45.5–100] mm/1st hour. Neutrophilic leukocytosis was observed in most of the patients. Anemia and hypertransaminasemia were also frequently reported, comprising 50% and 54.2%, respectively.

A small proportion of patients were positive for ANA antibodies (*n* = 2), RF (*n* = 2), and ACPA antibodies (*n* = 1). None of them fulfilled the classification criteria for rheumatoid arthritis, systemic lupus erythematosus, or connective tissue diseases.

### 3.2 Treatment and clinical outcomes

Almost all patients received oral glucocorticoids as initial therapy. Non-steroidal anti-inflammatory drugs (NSAIDs) and colchicine were also administered in 58.3% and 29.2% of the patients, respectively.

Conventional disease-modifying antirheumatic drugs (cDMARDs) were used in 17 (70.8%) patients. Methotrexate (MTX) was the first-line agent in all cases. Eight patients experienced complete clinical response, seven patients had only partial improvement, and two patients did not respond. Two refractory patients were switched to azathioprine (AZA) and leflunomide. These patients did not respond to these drugs either.

Biologic therapy was required to achieve remission in 13 (54.1%) patients. Three patients were naive to cDMARDs. Anakinra was the most used (*n* = 8), followed by anti-TNF agents (*n* = 5), anti-IL-6R (*n* = 6), and canakinumab (*n* = 3).

### 3.3 NGS analysis

DNA was extracted from blood samples of the 24 AOSD patients. Genetic variants were observed in five (20.8%) patients. None of them were classified as pathogenic based on the ACMG criteria (25). Variants of uncertain significance (VUS) were identified in two patients in NOD2 (c.2104C>T and c.2251G>A), in one patient in TNFRSF1A (c.224C>T), in another patient in TNFAIP3 (c.1939A>C), and in another one in SCN9A (c.2617G>A). The clinical features and therapeutic responses of these patients are shown in **Table 2**.

**Table 2:** Clinical manifestations and course of the disease in AOSD patients with genetic variants.

	VUS	Demographic and clinical features	Therapeutic response
Patient 1	*NOD2 (c.2104C>T)*	Male/48 years Fever, erisipela-like rash, arthralgia, lymphadenopathy, abdominal pain, asthenia	Partial response to GC, NSAIDs, and colchicine Complete response with etanercept
Patient 2	*NOD2 (c.2251G>A)*	Female/49 years Fever, macular rash, arthralgia, odynophagia, severe sicca syndrome, asthenia, constitutional syndrome	Partial response to GC and MTX. Complete response to anakinra but it was discontinued due to allergic reaction and switched to canakinumab, achieving clinical remission
Patient 3	*TNFRSF1A (c.224C>T)*	Male, 18 years Fever, erisipela-like rash, odynophagia, pleuropericarditis, lymphadenopathy, abdominal pain, asthenia, constitutional syndrome, testicular pain	Partial response to GC, NSAIDs, colchicine, MTX, and anakinra Infusional reaction with TCZ Complete response with canakinumab
Patient 4	*TNFAIP3 (c.1939A>C)*	Female, 60 years Fever, macular rash, arthritis, odynophagia, asthenia	Complete response with GC
Patient 5	*SCN9A (c.2617G>A)*	Female, 19 years Fever, arthralgia, arthritis, pleuritis, lymphadenopathy, splenomegaly, macrophage activation syndrome, leukopenia, tetraperesis due to leukoencephalopathy	Partial response to GC. Refractory to NSAIDs, MTX, AZA, LFN, CFM, and etanercept Complete response with anakinra

## 4 Discussion

Molecular genetic techniques are becoming increasingly essential tools for the diagnosis of SAIDs. However, their role in the diagnostic algorithm of AOSD still remains limited. Previous studies assessing the potential genetic variants in AOSD have only assessed single coding regions of a few genes using Sanger sequencing (5–9). However, NGS panels are becoming the genetic technique of choice because they allow rapid and simultaneous analysis of the complete coding sequence of several SAID-related genes (15). In addition, increasing the NGS gene panel has been related to a higher diagnostic rate in autoinflammatory diseases (26). To the best of our knowledge, our study is the first to assess the role of NGS panels in AOSD patients.

We found that 20.8% of our 24 AOSD patients were carriers of genetic VUS in genes related to other SAIDs, including NOD2, TNFRSF1A, TNFAIP3, and SCN9A. No pathogenic genetic variants were identified. **Table 3** summarizes previous reported data of genetic studies in AOSD patients. The prevalence of VUS varies widely across populations. Our results are in line with those reported in a Caucasian population ranging from 15% to 27.8% (9). However, Asian studies found a higher frequency of VUS in MEFV. In this regard, a prevalence of 53.1% and 63.3% of VUS in MEFV was reported in Korean and Japanese AOSD patients, respectively (5, 8). It is worth mentioning that none of our patients carried VUS in MEFV.

**Table 3:** Literature review of periodic fever syndrome genetic variants identified in AOSD patients.

	*Kim et al. (2013)* (5)	*Cosan et al. (2013)* (6)	*García-Melchor et al. (2014) (* 7)	*Nonaka F. et al. (2014)* (8)	*Sighart et al. (2018)* (9)	*Present study (2024)*
AOSD diagnosis	Yamaguchi criteria	Yamaguchi criteria	Yamaguchi criteria	Yamaguchi criteria	Yamaguchi criteria	Yamaguchi and/or Fautrel criteria
Number of patients/country	96/Korea	20/Turkey	18/Spain	49/Japan	40/Germany	24/Spain
Genotyping technique	Sanger sequence analysis	Sanger sequence analysis	Sanger sequence analysis	Sanger sequence analysis	Sanger sequence analysis	Targeted NGS panel
Genes tested (specific mutations)	*MEFV* (E148Q, P369S, M680I, V726A, M694V)	*MEFV* (M694V, E148Q, V726A, M680I) *TNFRSF1A* (exons 2–3 and exons 4–5)	*NLRP3* (exon 3) *NOD2* (exon 4)	*MEFV* (exons 1, 2, 3, and 10)	*MEFV* *TNFRS1A* *NLRP3* *MVK* *NOD2* (coding exons and flanking intronic sequence)	*ACP5, ADA2, ADAM17, ADAR, ARPC1B, C17orf62, CARD14, CIB1, COPA, DNASE2, IKBKG, IL-10, IL-10RA, IL-10RB, IL-1RN, IL-36RN, JAK1, JAK3, LACC1, LPIN2, LRP5, MEFV, MVK, NLRC4, NLRP1, NLRP12, NLRP3, NOD2, OTULIN, PLCG2, POLA1, POMP, PROC, PSMA3, PSMB10, PSMB3, PSMB4, PSMB8, PSMB9, PSMG2, PSTPIP1, RBCK1, RC3H1, RIPK1, SLC29A3, TMEM173, TNFAIP3, TNFRSF1A, WDR1*
Patients with genetic variants, *n* (%)	51 (53.1%)	3 (15%)	5 (27.8%)	31 (63.3%)	6 (15%)	5 (20.8%)
VUS (*n*)	*MEFV*: P369S (7), E148Q (44)	*MEFV*: M694V (1), M6801 (1), E148Q (1)	*NLRP3*: V198M (1), Q703K (1) *NOD2*: R702W (2), R791Q (1)	*MEFV*: *M694I* (2), *G632S* (1), *P369S* (6), *R408Q* (5), *L110P* (8), *E148Q* (29), *E4K* (2)	*MEFV*: A653H (1), A744S (1), G62T (1) *TNFRSF1A*: C81T (1), I199A (1) *NLRP3*: V200M	*TNFRSF1A*: P75L *NOD2*: R675W, E751K *TNFAIP3*: T647P *SCN9A*: G884S

**Figure 2:**
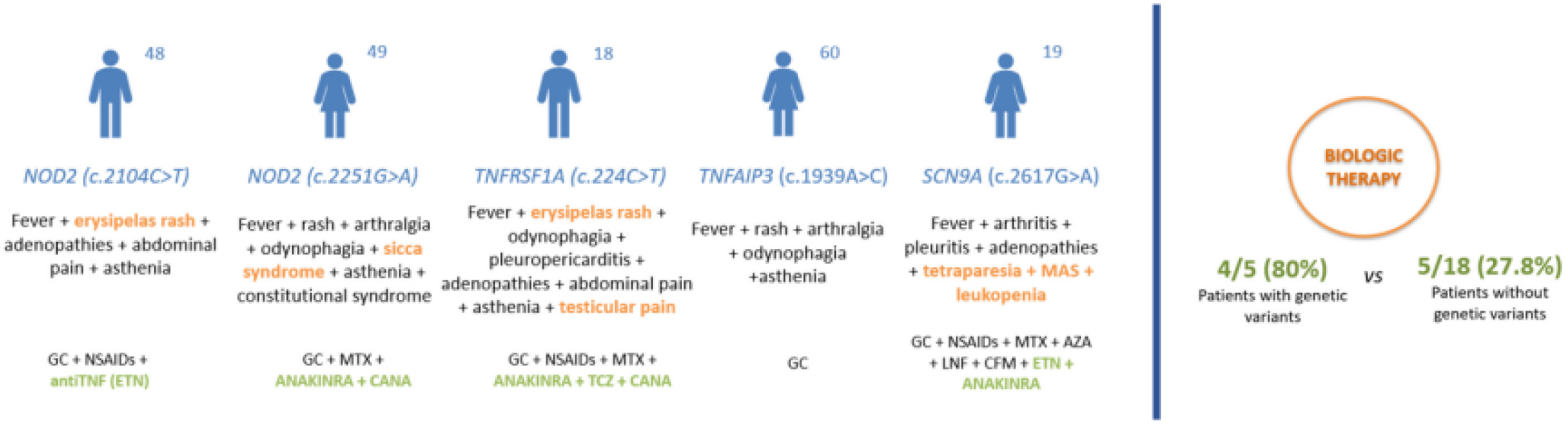
Clinical features and therapeutic response in AOSD patients with genetic variants. AZA, azathioprine; CANA, canakinumab; GC, glucocorticoid; CFM, cyclophosphamide; ETN, etanercept; LFN, leflunomide; MAS, macrophage activation syndrome; MTX, methotrexate; NSAIDs, non-steroidal anti-inflammatory drugs; TCZ, tocilizumab.

The clinical implications of carrying genetic VUS on severity and therapeutic response in AOSD patients are still under investigation. Our two patients with NOD2 variants did not present atypical manifestations but were refractory to conventional DMARDs and required biologic therapy to control disease activity. The patient carrying a genetic VUS in TNFRSF1A experienced testicular pain, which is not frequent in AOSD, and was refractory to different lines of DMARDs requiring canakinumab to achieve complete remission. The patient with SCN9A VUS had atypical severe neurologic manifestations and responded poorly to different cDMARDs and biological therapy until anakinra was initiated. In contrast, the patient with the TNFAIP variant did not show atypical symptoms and achieved a complete response with glucocorticoids alone (**Table 2**; **Figure 2**).

Taking into account our whole cohort of 24 AOSD patients, 4 out of 5 patients with genetic variants required biologic therapy, while only 5 of 18 patients without genetic variants needed biologics due to cDMARD refractoriness. These findings suggest that AOSD patients with genetic variants might be prone to a more severe and refractory course of the disease. In this regard, Nonaka et al. (8) found that MEFV VUS carriers were more likely to present with a polycyclic phenotype and require biological therapy to control inflammatory disease activity. Along the same lines, Sighart et al. (9) reported that two-thirds of patients with VUS in MEFV experienced a severe course of the disease and two patients in whom TNFRSF1A genetic variants were detected required bDMARDs.

Recent studies have identified and validated four distinct patient clusters in AOSD, as well as three disease-related endotypes, each characterized by different immune cell profiles (27–29). In our study, among the five patients carrying genetic VUS, one patient (with a *TNFRSF1A* VUS) could be classified into cluster 1 or the “juvenile/transitional” group, one patient (with a *TNFAIP3* VUS) into cluster 2 or the “uncomplicated” group, two patients (with *NOD2* VUS) into cluster 3 or the “hyperferritinemic” group, and one patient (with *SCN9A* VUS) into cluster 4 or the “catastrophic” group. Taking this into account, future studies focused on the distribution of VUS across the different clusters would be highly valuable, as it may help elucidate their role in driving variations in clinical presentation, prognosis, and treatment response.

This study has potential limitations, including a small sample size and regional specificity (northern Spain). Additionally, we recognize that the use of a targeted gene panel may not capture all relevant variants, and the VUS identified were not classified as pathogenic, which complicates their interpretation. Despite these limitations, we consider that the use of NGS in our cohort of patients allowed us to detect genetic VUS that were associated with atypical clinical manifestations and refractoriness to conventional therapy. Based on our experience, we advocate for the use of NGS in all AOSD patients to exclude other SAIDs, facilitate the identification of those who may present with atypical manifestations, and improve the detection of patients likely to exhibit poor treatment response. Future collaborative international studies are particularly needed to enlarge the patient sample and encompass broader regional areas. Whole-exome sequencing will also help to identify more coding variants.

In conclusion, our findings support that NGS could be of value in AOSD patients. NGS was useful to rule out the presence of pathogenic genetic variants related to other SAIDs and to detect VUS that might be associated with a more severe and refractory course of the disease. Further studies are needed to establish stronger associations. The detection of VUS may help to identify patients at risk for atypical and severe manifestations and poor response to conventional therapy.

## Data Availability

The original contributions presented in the study are included in the article/supplementary material. Further inquiries can be directed to the corresponding author.

## Author’s note

A detailed study protocol was developed prior to commencing the research, which was strictly followed and reviewed to avoid biases in the study design and interpretation of results.
